# You Shall Not Pass: MX2 Proteins Are Versatile Viral Inhibitors

**DOI:** 10.3390/vaccines11050930

**Published:** 2023-05-03

**Authors:** Gilberto Betancor

**Affiliations:** Instituto Universitario de Investigaciones Biomédicas y Sanitarias (IUIBS), Universidad de Las Palmas de Gran Canaria, 35016 Las Palmas de Gran Canaria, Canary Islands, Spain; gilberto.betancor@ulpgc.es

**Keywords:** virus, infection, MX2, inhibition, antiviral, HIV-1

## Abstract

Myxovirus resistance (MX) proteins are pivotal players in the innate immune response to viral infections. Less than 10 years ago, three independent groups simultaneously showed that human MX2 is an interferon (IFN)-stimulated gene (ISG) with potent anti-human immunodeficiency virus 1 (HIV-1) activity. Thenceforth, multiple research works have been published highlighting the ability of MX2 to inhibit RNA and DNA viruses. These growing bodies of evidence have identified some of the key determinants regulating its antiviral activity. Therefore, the importance of the protein amino-terminal domain, the oligomerization state, or the ability to interact with viral components is now well recognized. Nonetheless, there are still several unknown aspects of MX2 antiviral activity asking for further research, such as the role of cellular localization or the effect of post-translational modifications. This work aims to provide a comprehensive review of our current knowledge on the molecular determinants governing the antiviral activity of this versatile ISG, using human MX2 and HIV-1 inhibition as a reference, but drawing parallelisms and noting divergent mechanisms with other proteins and viruses when necessary.

## 1. Introduction

One of the first cellular defenses against viral infection is the production of interferons (IFNs). IFNs are cytokines with pro-inflammatory and immunomodulatory activities, which induce the upregulation of IFN-stimulated genes (ISGs) [1,2,3]. The notion that IFNs, and, especially, type I IFN, are potent inhibitors of viral replication has been known for over 60 years [4,5,6,7], reviewed in [8]. However, the IFN response is not specific and hundreds of ISGs are expressed independently of the pathogen triggering IFN production. Therefore, it is advantageous for the cell to produce ISGs with broad antiviral activity. A well-known ISG restricting many different viruses is the myxovirus resistance 1 (MX1) protein. MX1, also called MXA in humans and denoted Mx1 in rodents and organisms other than mammals, is a potent inhibitor of DNA and RNA viruses including influenza A virus (IAV), La Crosse encephalitis virus, Thogoto virus, or hepatitis B virus (HBV), but not human immunodeficiency virus 1 (HIV-1) [9,10,11,12,13,14]. However, it has been shown that treatment of HIV-1-infected individuals with IFNα produces a reduction in the frequency of viral isolation by culture and a decrease in patients developing AIDS compared to a placebo group [15]. This unequivocally points to the presence of ISGs capable of inhibiting HIV-1. 

**Figure 1:**
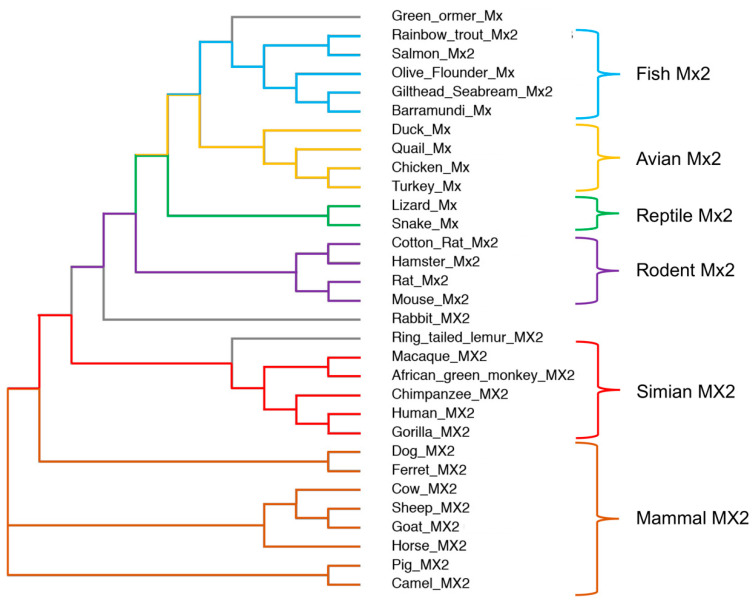
Phylogenetic neighbor-joining tree of MX2 proteins. MX2 amino acid sequences were obtained from the GeneBank, and multiple sequences alignment was calculated using the Clustal Omega tool from EBML-EBI (https://www.ebi.ac.uk/Tools/msa/clustalo/, accessed on 6 February 2023) and the ClustalW output format. MX2 proteins are divided into 6 groups based on their similarity, differentiating between fish, avian, reptile, rodent, simian, and other mammalian MX2s.

Most mammals bear a second MX gene, called *MX2*, which arose from an ancient duplication event. This has resulted in two different lineages, the MX1-like and the MX2-like lineage. Interestingly, rodent Mx2 is similar to human MX2 (hMX2/MXB), only by name, since the rodent *Mx2* gene is a paralog of *Mx1*, while the human-like *Mx2* was lost during evolution. Similarly, fish, birds, and reptiles do not have a human-like *Mx2* gene (Figure 1). Based on this, it should be, therefore, noted that proteins such as rat Mx2, mouse Mx2, or any fish Mx protein are not orthologs of hMX2 or any other mammalian MX2, but orthologs of hMX1 and, therefore, have an hMX1-like antiviral activity, inhibiting rhabdoviruses, nodaviruses, birnaviruses, bunyaviruses, or orthomyxuviruses [16,17,18,19,20,21,22,23,24,25,26,27,28]. hMX2 was first characterized as an ISG potently upregulated by type I IFN (IFNα and IFNβ) induction, and without antiviral activity against IAV or vesicular stomatitis virus (VSV) [29,30]. This early failure to inhibit viral infection stuck with hMX2 for over 20 years, being largely considered devoid of antiviral activity. However, this notion dramatically changed in 2013, when hMX2 was first revealed as a potent inhibitor of HIV-1 [31,32,33], as reviewed in [34]. 

This review aims to comprehensively summarize our current understanding on mammalian MX2 biology and antiviral activity, with a special emphasis on HIV-1 inhibition by hMX2.

## 2. Breadth of MX2 Antiviral Activity

After a decade of research, the list of viruses restricted by hMX2 has steadily grown, including now distinct viral families such as herpesviruses or flaviviruses. In addition, MX2 proteins from other organisms, such as equine MX2 or porcine MX2, have been evaluated for their antiviral activity. From all these studies two main conclusions can be drawn: (i) different viral families are inhibited by the same MX2 protein, highlighting the important role of this protein on the IFN response to infection, and (ii) the viral and MX2 determinants governing this inhibition are diverse, with the only commonality of the direct interaction between MX2 and a viral component. Therefore, hMX2 interacts with the capsid (CA) of HIV-1 [35,36,37], the NS5A protein of hepatitis C virus (HCV) [38], or with tegument-free capsids from herpes simplex virus 1 (HSV-1) [39,40]. The effect of this interaction is mostly unknown, with different works proposing either stabilization of HIV-1 CA [36], disassembly of herpesviruses HSV-1, HSV-2, and varicella zoster virus (VZV) capsids [40], or mislocalization of HCV NS5A from the endoplasmic reticulum [38]. However, in all cases, hMX2 inhibition seems to occur shortly after infection by inhibiting RNA replication of HCV and hepatitis B virus (HBV) [38,41] or by blocking the nuclear import of viral replication complexes in the case of herpesviruses and retroviruses [31,32,33,40,42,43,44,45].

A summary of all existing data linking MX2 proteins with the viruses they restrict can be found in Table 1.

## 3. MX2 Structure

**Figure 2:**
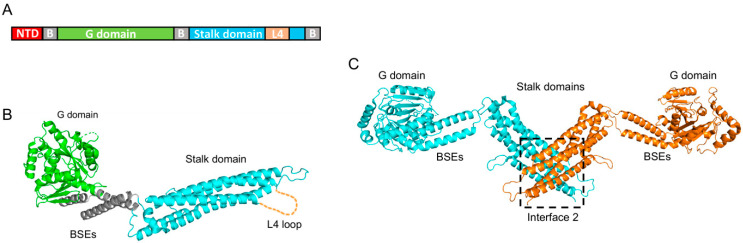
Structure of hMX2. (**A**) Scheme of hMX2 domain organization, indicating the amino-terminal domain (NTD), bundle signaling elements (B), GTPase domain (G domain), stalk domain, and the L4 loop (L4). (**B**) Crystal structure of the hMX2 monomer from Fribourgh et al. [35] (PDB entry 4WHJ), in cartoon representation, and following the same color code as (**A**). The L4 loop (missing in the original structure) is represented by a dashed line. (**C**) Crystal structure of the hMX2 dimer in cartoon representation with one monomer in cyan and the other in orange. The dimerization interface (interface II) is highlighted (PDB entry 4WHJ).

hMX1 and hMX2 proteins belong to the family of large dynamin-like GTPases and share a 63% sequence identity at the amino acid level. Due to its earlier recognition as an important viral restriction factor, hMX1’s structure was resolved over a decade ago [59,60], as reviewed in [61]. These structural works show that hMX1 comprises an amino-terminal domain (NTD), followed by a GTPase domain (G domain) and a carboxy-terminal stalk domain (which harbors an unstructured loop, called the L4 loop), all connected by a tripartite bundle signaling element (BSE) (Figure 2A). Further biochemical studies have shown that the stalk domain is critical for oligomerization [59], and that conformational changes that originated in the G domain upon GTP binding and hydrolysis are transmitted to the stalk domain via the BSEs [60]. 

Owing to their similarity, the hMX2 structure resembles that of hMX1. Therefore, G domain, BSEs, and stalk domain are distributed analogously (Figure 2B) [35,62]. However, hMX2 has a longer NTD compared to hMX1 (91 vs. 43 amino acids, respectively). Unfortunately, efforts to obtain the crystal structure of the NTD have been unsuccessful, most likely due to the high mobility of this domain. Therefore, Fribourgh and colleagues crystallized a truncated form of hMX2 missing the first 83 residues [35]. They found that while hMX1 and hMX2 individual domain structures are very similar, with a root-mean-square deviation (rmsd) of around 1 Å, the overall rmsd between them is over 6.4 Å, an indication of large differences in domain orientation. Nevertheless, and akin to hMX1, two hMX2 monomers assemble forming an antiparallel dimer, where the stalk domain of each protomer interacts with the stalk domain of the opposite one (Figure 2C). Cryo-electron microscopy studies carried out by Alvarez and colleagues further demonstrated that hMX2 assembles into higher-order structures where six hMX2 dimers form a rung with G domains and NTDs facing outside, and contain the stalk domains in the inner rung space [62]. These 12-mer rungs interact with each other, building long tubular structures on a one-start right-handed helix conformation. An interesting observation by these authors was that such hMX2 tubes can be disassembled by the addition of GTP, but not analogs GMP-PCP or GTP-γ-S analogs, or GDP.

Comprehensive structural and biochemical characterizations of hMX2 have determined the existence of four different interaction interfaces. Interface II, located on the stalk domain, is essential for dimerization and includes residues Met574 or Tyr651, whose mutation renders monomeric forms of hMX2 [35,62,63,64]. Interfaces I and III connect hMX2 dimers and contain residues, such as Phe420 and Lys693, and Glu484 and Glu491, respectively. Finally, hMX2 contains a vertical interface, connecting adjacent rungs, denoted interface IV and involving residues Lys250, Pro284, and Glu285 from one rung and Arg674, Trp677, and Gln680 from the other rung. The mutation of residues from interfaces I, III, or IV do not affect the ability of hMX2 to form dimers, but greatly reduces oligomerization into higher-order structures, sometimes resulting in a decrease in antiviral activity (as seen in Section 5).

Table 2 contains a summary of all the hMX2 mutations discussed in this article and their effect on HIV-1 inhibition.

## 4. Role of MX2 NTD

hMX2 exists as two isoforms of ~76 and ~78 KDa, both IFN-inducible, due to the presence of an alternative start codon at position 26 [67,68]. This results in two proteins with NTDs of 66 and 91 amino acids, respectively. It was early found that only the long isoform inhibits HIV-1 infection, probing the essential role of the first 25 amino-terminal residues in viral restriction [43,57]. The following works also demonstrated that all the requirements for hMX2 anti-HIV-1 activity are contained on the NTD, so the transfer of this domain to proteins unable to restrict HIV-1 (such as hMX1 or the MLV restriction factor Fv1b) resulted in fully antiviral chimeras [36,57,65]. Inhibition of HCV and herpesviruses also requires the entire NTD, and it has been shown that murine cytomegalovirus (MCMV) and HSV-1 are not restricted by hMX2 short isoform (hMX2∆1-25) [38,40,42,45]. In contrast, HBV is inhibited by hMX2∆1-25 [41], pointing to a distinct antiviral mechanism. Surprisingly, equine MX2 (eMX2) potently inhibits EIAV, HIV-1, HIV-2, FIV, and SIV, and also blocks the nuclear import of viral replication complexes, while lacking the NTD region complementary to hMX2’s first 25 amino acids [50,51]. This striking difference points to a restriction mechanism orchestrated by determinants not contained on the first NTD 25 residues, demonstrating a remarkable flexibility of MX2 proteins.

Some important features for the NTD antiviral function have been identified. A stretch of three arginines spanning positions 11 to 13 is critical for the HIV-1 inhibitory activity of hMX2 and the mutation of these residues to alanine or lysine produced inactive proteins [64]. The specific function of these residues was not clear until their role in HIV-1 CA interaction was uncovered. HIV-1 CA is composed of many copies of the capsid (or p24) protein, assembled into hexamers and pentamers, building a fullerene cone-shaped vessel that shields the viral RNA and enzymes from the cytosolic environment [69]. Point mutations in CA, such as N74D, P90A, or G208R, can yield MX2-insensitive viruses [31,32,33,49,70]. Importantly, all these CA point mutants are bound by hMX2, as shown by interaction experiments [35,36,37,48,71], highlighting a pivotal feature of HIV-1 inhibition: CA binding is necessary but not sufficient for restriction to happen. Further biochemical and structural studies found that only assembled forms of CA interact with hMX2 [35]. By using sophisticated CA assemblies exposing individual surfaces found in mature CAs, Summers and colleagues showed that hMX2 binds to the three-fold inter-hexamer interface on HIV-1 CA [72]. The same investigators followed on, showing that arginines 11-13 on the hMX2 NTD bind to this region, and that mutant R11-13A is unable to maintain the interaction, explaining the lack of antiviral activity of this protein [73]. Further confirmation of the importance of the CA tri-hexamer interface on HIV-1 inhibition is shown by the selection of mutations, such as P207A, G208R, and T210K, located on this same CA surface, on HIV-1 in vitro evolution experiments carried out in the presence of hMX2 [49]. 

In contrast, the triple-arginine motif is not required for HSV-1 inhibition [47] and, therefore, is not involved in capsid interaction, as it is shown by the ability of hMX2∆1-25 to bind to HSV-1 capsids [40].

## 5. MX2 Oligomerization

As discussed before, MX proteins form large oligomers owing to the presence of different interaction interfaces. By analyzing the antiviral activity of interface II mutant proteins such as M574D or Y651D, it was shown that monomeric forms of hMX2 are not capable of HIV-1 restriction [35,62,63,64]. Concomitantly, chimeric proteins bearing the NTD of MX2 only inhibited HIV-1 when in a dimeric or higher-order oligomerization state [58]. However, oligomerization to structures larger than dimers seems to enhance the antiviral activity of hMX2, since interface I, III, and IV mutants often show weaker inhibition of HIV-1 infection compared to wild-type proteins [35,62,63,64,74].

Akin to HIV-1 inhibition, HSV-1 infection is not restricted by the monomeric mutant M574D [47]. In contrast, inhibition of HBV requires oligomers larger than dimers [41], which again highlights the various mechanisms employed by hMX2 to block viral infection.

## 6. G domain and GTPase Activity

hMX1 exploits its GTPase activity to inhibit viral infection [75,76], and this led to an analysis of the antiviral activity of GTPase-deficient hMX2 mutants. Residue Lys131 is involved in GTP binding, while Tyr151 participates in the catalytic step, producing the hydrolysis of GTP into GDP and a phosphate group. Therefore, mutant K131A is unable to bind GTP, while T151A binds GTP but cannot produce its hydrolysis. However, both proteins maintain at least some antiviral activity against HIV-1 [31,32,43,48,77]. In contrast, eMX2 seems to require both GTP binding and hydrolysis, since mutants K127A (equivalent to hMX2 K131A) and T147A (equivalent to hMX2 T151A) are strongly compromised in their antiviral activity, only mildly inhibiting HIV-1, but not EIAV [50].

The role of GTPase activity in herpesvirus inhibition is less clear. Therefore, while all available data agree on the inability of K131A to inhibit MCMV and HSV-1 infection, there is not a clear picture for the requirement of GTP binding. Schilling and colleagues found that mutant T151A inhibited HSV-1 and MCMV replication (an alpha- and betaherpesvirus, respectively) but not infection by murine gamma herpesvirus 68 (MHV68, a gammaherpesvirus), pointing to different hMX2 requirements for the inhibition of distinct herpesviruses [47]. However, other studies have found that T151A is unable to inhibit HSV-1 [45] or MCMV [42]. At least in the case of HSV-1, these data are supported by the finding that although T151A hMX2 binds to viral capsids, this does not result in their shredding [40]. Similar to the findings of Schilling and colleagues, HBV inhibition seems to require GTP binding but not hydrolysis [41]. 

While GTPase activity is not necessary for HIV-1 inhibition, the G domain itself plays an important role. Interaction data generated using truncated forms of hMX2 lacking the whole NTD found that these proteins still bind to HIV-1 CA [35,37], supporting data showing that proteins bearing a mutated NTD triple-arginine motif were also able to bind HIV-1 CAs in vitro [37]. It was then found that the G domain of hMX2 contains an HIV-1 CA binding motif involving residues Gly184, Asn260, and Gln351. HIV-1 replication experiments showed that G domain interaction enhances the antiviral activity of the protein [37]. A more important finding was that the G domain-CA interaction allows hMX2∆1-25 to outcompete the binding of the long isoform to CA, producing a drop in antiviral potency. In fact, the overexpression of hMX2∆1-25 damped the IFN-imposed HIV-1 restriction on parental U87-MG cells but not on cells where hMX2 was depleted, indicating that hMX2∆1-25 negatively regulates the long isoform antiviral activity and, concomitantly, the type I IFN block to HIV-1 infection [37]. While the underpinning mechanism behind the regulatory role played by the hMX2 short isoform is unknown, it has been proposed it could be downmodulating deleterious effects of the full-length form on cell metabolism.

The existence of multiple interacting points between hMX2 and CA highlights the complexity of the inhibitory mechanism. Proof of the physiological importance of the hMX2-CA interaction is provided by a recent work reporting how viruses isolated from HLA B27/B57^+^ elite controller patients (infected individuals able to control viral replication without antiretroviral treatment) bear CA mutations such as G116A, reducing susceptibility to hMX2 inhibition [78]. In fact, an interesting model has been proposed wherein the CA conformation, affected by the interaction with different cellular factors during infection, dictates sensitivity to hMX2. Therefore, various CA point mutations conferring resistance to hMX2, such as P90A or N74D, would achieve so by modifying the flexibility/conformation of CA, rendering it unamenable for restriction, rather than by preventing the interaction of CA with other cellular factors [79].

## 7. Subcellular Localization

Intracellular staining shows that hMX2 decorates the nuclear envelope and forms cytoplasmic puncta [31,32,43,58,80]. The first 25 amino acids are again essential for nuclear envelope localization since the short isoform is only present in the cytoplasm. However, it is possible that other regions from the NTD or even different domains from hMX2 modulate its cellular distribution. In fact, the transfer of the first 25 residues of hMX2 to an unrelated protein leads to its nuclear accumulation, instead of decorating the nuclear envelope [64,81]. Immunofluorescence microscopy analysis of mutated hMX2 proteins has pinpointed Lys20 and Tyr21 as part of the NTD motif directing hMX2 to the nuclear envelope [66]. Missing the equivalent hMX2 first 25 residues, eMX2 accumulates mostly in the cytoplasm, with only a small fraction decorating the nuclear envelope. However, retroviral infection seems to relocate a larger fraction of the protein to the nuclear envelope [51].

It is still unclear how hMX2 localization affects viral inhibition, mostly due to the difficulty of isolating this individual feature from others. Therefore, while deletion of the first 25 amino-terminal residues produces the cytoplasmic accumulation of the protein, it also eliminates other pivotal modulatory activities of the NTD. Nevertheless, several studies have found a correlation between the extent of nuclear envelope accumulation and the potency of inhibition [43,74,82]. Therefore, type I IFN treatment of cells overexpressing hMX2 further accumulates the protein in perinuclear aggregates [50], hinting at nuclear-envelope-associated hMX2 being the physiologically relevant HIV-1 inhibitor. Another line of evidence supporting this model is the requirement of specific sets of nuclear pore proteins for antiviral activity [65,80,82] (as seen in Section 8). In contrast, others have shown that the K20A hMX2 mutant, with impaired ability to aggregate at the nuclear envelope, maintains its antiviral activity [66]. More intriguingly are the results obtained by Kane and colleagues, who found that a chimeric protein bearing amino acids 1-91 of hMX2 fused to a cellular protein, ARFAPTIN2, was fully antiviral while localizing only as cytoplasmic aggregates [81]. Finally, it has been proposed that both cytoplasmic and nuclear-envelope-associated hMX2 inhibit HIV-1. This conclusion is based on the observation that the K131A mutant has a markedly reduced nuclear envelope localization compared to the wild-type protein while still being able to inhibit HIV-1 replication [77]. In sharp contrast, the T151A mutant is mainly nuclear-envelope-associated but does not enhance viral restriction [43,48,77]. Interestingly, mutant T151D does boost antiviral activity while increasing nuclear envelope accumulation [48]. These data point to a complex balance between the amount of hMX2 present in the nuclear envelope, the binding or not of GTP, and the extent of the antiviral activity.

The available evidence for herpesvirus inhibition supports a picture akin to HIV-1, showing that the hMX2 short isoform is not antiviral [38,40,42,45]. However, whether this is due to being localized to the cytoplasm or to the lack of other necessary NTD functions is unknown. In contrast, published data show that cytoplasmic hMX2 (∆1-25) inhibits HBV [41]. HCV inhibition does require the nuclear accumulation of hMX2, but apparently no other NTD functionalities, because chimeric forms of hMX2(∆1-25) attached to the nuclear localization signal from simian virus 40 (SV40) large T antigen are able to inhibit viral replication [38].

## 8. Role of Other Cellular Proteins

One of the first CA mutants to be identified as hMX2-resistant was the P90A virus. Pro90 is located on an exposed CA loop named cyclophilin binding loop for being the interaction point of human cyclophilin A (CypA), a well-known HIV-1 cofactor [83,84]. This prompted the investigation of the role played by CypA in the antiviral activity of hMX2. Although some initial reports did not find CypA to be required for hMX2-mediated HIV-1 inhibition [49], it is now well accepted that CypA depletion or inhibition by cyclosporine A (CSA) negatively impacts the antiviral activity of hMX2 [33,79,81,85]. Although the mechanism of this dependence is not understood, we know that hMX2 does not bind to the same CA surface as CypA [36], and that the CypA requirement is time-dependent since adding CSA at 12 h post-infection no longer had an effect on hMX2 inhibitory activity [79]. The same authors found that the A92E mutant virus, which is insensitive to CypA depletion/CSA treatment, is inhibited by hMX2, proposing that hMX2 does not require CypA per se, but the effect CypA exerts on CA, somehow altering the CA conformation, making it sensitive to hMX2 inhibition.

In a yeast two-hybrid screening against a human leukocyte cDNA library, several nucleoporins (NUPs) and nuclear pore proteins were scored as interactors of hMX2. Further validation of this screening found that the depletion of nucleoporin 214 (NUP214), transportin 1 (TNPO1), and, especially, both together significantly reduced the antiviral activity of hMX2 in HeLa cells [82]. Importantly, dependence on these two nuclear pore proteins was confirmed in IFNα-treated primary CD4^+^ T cells and linked to reduced nuclear envelope accumulation of hMX2. Further support for the role of nuclear pore proteins on hMX2 antiviral activity can be found in an impressive work by Kane and colleagues, who systematically depleted every single nuclear pore protein and analyzed their effect on hMX2 inhibitory activity. These authors showed that hMX2 requires the participation of some NUPs (such as NUP98, NUP153, or NUP214) for its ability to restrict HIV-1 replication, but this varies with the cell line analyzed. They also found that NUP358 is required for the recruitment of hMX2 to the nuclear pore late in mitosis [81]. Finally, it has been proposed that the NUP358-hMX2 interaction reduces the colocalization of NUP358 with CA, ultimately impairing the nuclear import of HIV-1 replication complexes [86]. What is clear from all these works is that hMX2 exploits nuclear pore proteins to inhibit viral infection, with the interesting possibility of being able to use different subsets of NUPs, depending on the nature of the nuclear pore.

hMX2 has been proposed as a necessary player for the antiviral activity of HIV-1 restriction factor SAM domain and HD domain-containing protein 1 (SAMHD1). SAMHD1 inhibits HIV-1 infection in macrophages, resting CD4^+^ T cells, and dendritic cells by reducing the level of dNTPs available for reverse transcription [87,88]. By detecting a reduction in HIV-1 inhibition by SAMHD1 when hMX2 is depleted, the authors propose that hMX2 is required for the antiviral activity of SAMHD1 by an unknown mechanism not involving the interaction between both proteins or a modification of the ability of SAMHD1 to reduce dNTP levels [89]. Similarly, it is proposed that cleavage and polyadenylation factor 6 (CPSF6) work alongside hMX2 to inhibit HIV-1 infection. CPSF6 is a well-known CA interactor modulating the nuclear import of viral replication complexes [90]. A model has been suggested where CPSF6 cooperates with hMX2 to inhibit the association of NUP358 with CA, hence inhibiting the nuclear import of replication complexes. This is translated into increased infection on hMX2-expressing HeLa cells when CPSF6 is depleted with small interfering RNAs (siRNAs) [86]. However, this role of CPSF6 on hMX2 antiviral activity seems controversial because others have not recorded the same effect when depleting CPSF6 by short hairpin RNA (shRNA) treatment on HEK293T cells [79].

## 9. Post-Translational Modification of hMX2

A mass spectrometry screen directed to identify hMX2-associated factors resulted in the discovery of two proteins, named myosin phosphatase target subunit 1 (MYPT1) and protein phosphatase 1 catalytic subunit-β (PPP1CB), both members of a holoenzyme called myosin light chain phosphatase (MLCP), a serine/threonine phosphatase [65]. It was then shown that the depletion of both proteins strongly reduced the HIV-1 inhibitory activity of hMX2. This finding led to the identification of several hMX2 residues subjected to phosphorylation. Particularly, a stretch of three serines, in positions 14, 17, and 18, was found to be essential for the antiviral activity of hMX2 because, their phosphorylation (or mutation to phosphomimetic aspartic acid), blocked the interaction between CA and hMX2 NTD. Importantly, IFNα treatment reduced the phosphorylation of this motif, hence “turning on” the antiviral activity of hMX2 [65]. 

Further investigation revealed several other hMX2 positions phosphorylated. The mutation of most of these residues to alanine (blocking the possibility of phosphorylation) or to aspartic acid did not affect hMX2 antiviral activity [48]. However, some of them strongly reduced it (such as T334D or T343A), while others enhanced it. This is the case of S28D, T151D, and T343D. Moreover, the combination of these residues produced an additive effect, further enhancing antiviral activity. Even more surprising was the finding that hypermorphic hMX2 variants S28D, T151D, and, especially, S28D/T151D show antiviral activity against CA mutant viruses resistant to hMX2 (such as P90A or T210K) and even other retroviruses, including EIAV and MLV. 

## 10. Other hMX2 Activities

hMX2 has been shown to inhibit the nuclear import of non-viral cargo. Therefore, hMX2 blocks the nuclear translocation of a chimeric GFP-LacZ protein attached with nuclear localization signals (NLSs) from C-MYC, DDX21, KNS, and hMX2 itself [81]. Interestingly, at least in some cases, this inhibitory activity is relieved by phosphorylation. Therefore, the nuclear import block of KNS NLS-GFP-LacZ is reduced when serines 14, 17, and 18 are mutated to phosphomimetic aspartic acid [65]. While the consequences of the hMX2-imposed nuclear import block are unknown, it could delay cell cycle progression, as has been proposed [80], or it could restrict the import of some transcription factors, hence modulating the transcriptional landscape of the cell.

hMX2 binds to the mitochondria membrane, and this is especially apparent in hepatoma cell lines, such as Hep3B or Huh7, and human primary hepatocytes where MX2 basal levels are high [91]. Interestingly, this association seems to require the first 25 residues from hMX2 since the short isoform is not found in mitochondrial membranes. hMX2 affects the size and shape of mitochondria, with depletion of the protein also leading to the loss of the mitochondrial genome (nucleoid), which is expelled in the cytoplasm [91].

Lastly, hMX2 has been proposed to restrict HIV-1 infection by an alternative mechanism involving the inhibition of the viral Rev protein [92]. According to this model, hMX2 inhibits the nuclear translocation of Rev, an activity that requires the first 25 amino acids but not protein dimerization since the M574D mutant phenocopies the wild-type protein. This results in a reduction in Rev-dependent HIV-1 Gag expression. The proposed mechanism involves a hMX2-mediated reduction in Rev association with TNPO1. This can be circumvented by the P31L Rev mutant, which does not require TNPO1 to traverse the nuclear membrane and is resistant to hMX2 inhibition [92].

## 11. Future Perspectives

The number of viruses inhibited by hMX2 is growing fast (see Table 1). However, it is very likely we only know a small fraction of them. This is best illustrated by the presence of residues subjected to strong diversifying selection not involved in the restriction of any of the presently known hMX2-inhibited viruses, indicating the presence of other hMX2-targeted pathogens not yet identified [93]. Nevertheless, many questions remain to be answered. For example, we still do not have a clear idea of how hMX2 inhibits HIV-1 infection, with only one work arguing it stabilizes the viral capsid, preventing uncoating [36]. Advanced microscopy approaches, able to track single viral particles and monitor the disassembly of viral CAs, will provide important insights into the antiviral mechanism of hMX2. 

Similarly, we still do not know exactly what cellular fraction of hMX2 is responsible for viral inhibition, with both (cytoplasmic and nuclear envelope) having some role ascribed. A comparison between hMX2 and eMX2 could answer this question since both proteins seem to inhibit HIV-1 infection through a similar mechanism while showing rather different cellular distribution. 

Likewise, while we know the importance of hMX2 phosphorylation for its anti-HIV-1 activity we do not know if other hMX2-inhibited viruses are similarly affected. It is possible to envision a scenario where differently phosphorylated forms of hMX2 inhibit different subsets of viruses.

Finally, it would be important to understand the non-antiviral activities of MX2, such as mitochondria metabolism regulation or the inhibition of nuclear import, because this could inform us of the not-yet-appreciated antiviral features of MX2 or, at a minimum, gives us some insight into the role played by this protein on cell metabolism. 

What is clear is that the antiviral mechanism/s of MX2 is/are complex, with several protein features involved in their regulation, granting further research on this molecular Swiss army knife.

## Figures and Tables

**Table 1:** Mammalian MX2 proteins and the viruses they inhibit.

MX2	Virus Family/Subfamily	Virus	Technique	Ref
Human	*Hantaviridae*	Hantavirus	PO	[44]
	*Rhabdoviridae*	Vesicular stomatitis virus (VSV)	PO	[45,46]
	*Hepadnaviridae*	Hepatitis B virus (HBV)	PD/PO	[41]
	*Flaviviridae*	Hepatitis C virus (HCV)	PD/PO	[38]
		Japanese encephalitis virus (JEV)	PO	[38]
		Dengue virus	PO	[38]
	*Alphaherpesvirinae*	Herpes simplex virus 1 (HSV-1)	PD/PO	[39,40,45,47]
		Herpes simplex virus 2 (HSV-2)	PO	[40,45]
		Varicella zoster virus (VZV)	PO	[40]
	*Betaherpesvirinae*	Murine cytomegalovirus (MCMV)	PO	[42,47]
	*Gammaherpesvirinae*	Kaposi-sarcoma-associated virus (KSHV)	PO	[45]
		Murine gamma herpesvirus 68 (MHV68)	PO	[47]
	*Orthoretrovirinae*	Human immunodeficiency virus 1 (HIV-1)	PD/PO	[31,32,33]
		Human immunodeficiency virus 2 (HIV-2)	PO	[32]
		Simian immunodeficiency virus (SIV)	PO	[31,32,43]
		Equine infectious anemia virus (EIAV)	PO *	[48]
		Murine leukemia virus (MLV)	PO *	[48]
		Mason–Pfizer monkey virus (MPMV)	PO	[49]
Equine	*Orthoretrovirinae*	HIV-1	PO	[50,51]
		HIV-2	PO	[50]
		SIV	PO	[50,51]
		EIAV	PD/PO	[50,51]
		SIV	PO	[50]
		MLV	PO	[50]
Porcine	*Arteriviridae*	Porcine reproductive and respiratory syndrome virus (PRRSV)	PO	[52]
	*Orthomyxoviridae*	Influenza A virus (IAV)	PO	[53]
	*Rhabdoviridae*	VSV	PO	[54]
Bovine	*Rhabdoviridae*	VSV	PO	[55]
	*Paramyxoviridae*	Caprine parainfluenza virus 3 (CPIV3)	PD/PO	[56]
African green monkey	*Arteriviridae*	PRRSV	PD/PO	[52]
	*Orthoretrovirinae*	HIV-1	PO	[49]
		MPMV	PO	[49]
Rhesus macaque	*Orthoretrovirinae*	HIV-1	PO	[49]
		MPMV	PO	[32,49]
Canine	*Orthoretrovirinae*	HIV-1	PO	[57,58]

PO—protein overexpression; PD—protein depletion; * using point mutant proteins.

**Table 2:** hMX2 mutations and their effect on HIV-1 inhibition.

Mutation	MX2 Domain	Antiviral Activity	Ref
∆1-25	NTD	Canceled	[32,43,57]
R11-13A	NTD	Canceled	[58]
S14, 17-18D	NTD	Canceled	[65]
K20A	NTD	Unaffected	[66]
Y21A	NTD	ND	[66]
S28D	NTD	Increased	[48,65]
K131A	G domain	Reduced	[31,32,43]
T151A	G domain	Unaffected	[32,43]
T151D	G domain	Increased	[48]
E285K	G domain	Unaffected	[62]
T334D	G domain	Decreased	[48]
T343A	G domain	Decreased	[48]
T343D	G domain	Increased	[48]
F420D	Stalk domain	Unaffected	[62]
E484K	Stalk domain	Decreased	[62]
E491K	Stalk domain	Unaffected	[62]
M574D	Stalk domain	Canceled	[35,63,64]
Y651D	Stalk domain	Canceled	[35,63,64]
W677D	Stalk domain	Unaffected	[62]
K693D	Stalk domain	Unaffected	[62]

NTD—amino-terminal domain; G domain—GTPase domain; ND—not determined.

## Data Availability

Not applicable.
